# The effect of background audio and audiovisual stimuli on students' autonomic responses during and after an experimental academic examination

**DOI:** 10.1002/brb3.3153

**Published:** 2023-07-31

**Authors:** İlker Balıkçı, Serdar Tok, Erdal Binboğa

**Affiliations:** ^1^ Faculty of Sports Sciences Manisa Celal Bayar University Manisa Turkey; ^2^ Faculty of Medicine, Department of Biophysics Ege University İzmir Turkey

**Keywords:** academic examinations, background television, classical music, heart rate variability

## Abstract

**Background:**

Due to the Covid‐19 pandemic lockdown during the online‐distant education period, certain students tended to combine their courses and homework with TV or social media news or other media content, such as classical music, including a wealth of audio and audiovisual stimuli. As the audio and audiovisual stimuli existing in a learning environment may affect students' autonomic nervous system (ANS) responses negatively, the present study aimed to monitor the impact of background TV, classical music, and silence on students' ANS activity represented by heart rate (HR), heart rate variability (HRV), blood volume amplitude (BVA), and skin conductance level (SCL) during and after an experimental academic examination.

**Method:**

Seventy‐six students were randomly allocated to background TV, classical music, or silence groups. The experiment with repeated measures design consisted of four consecutive periods: baseline, anticipation, challenge, and recovery, lasting 4 min each.

**Results:**

Within‐subject analyses indicated significant HRV decrement only in the background TV group. Regardless of the experimental groups, HR and SCL increased while BVA decreased during the task. In addition, the between‐subject analysis showed that the background TV group experienced significantly larger changes in HR and HRV parameters compared to the other experimental groups relative to their respective baseline measurements.

**Conclusions:**

Based on these results, we concluded that relative to classical music and silence, background TV, including audiovisual and verbal stimuli, extant in a learning environment might raise students' sympathetic activity. Further, classical music, without lyrics, may suppress the withdrawal of vagal activity and elevate the autonomic regulation capacity during the academic reading comprehension task. HRV is a more valid and reliable indicator of students' autonomic responses during a challenging academic task.

## Abstract 2

Background TV existing in a learning environment might give rise to a decrement in heart rate variability during an experimental academic examination. Classical music elevate the autonomic regulation capacity during the academic reading comprehension task. Heart rate, blood volume amplitude, skin conductance level, and heart rate variability might not be equally sensitive indices to environmental audio and audiovisual stimuli.

## 1 INTRODUCTION

Students face many challenges throughout their academic careers, such as examinations, harsh course content, and relations with teachers. The learning environment, which includes various dimensions such as the school buildings themselves, classrooms, the materials used for instruction, and interactions between students and teachers, can also be another severe challenge for students and might impact several variables. Previous research documented that the learning environment might affect certain outcome variables such as self‐efficacy (Dorman, 2001), coping style (Thuen et al., 2007), critical thinking (Jou et al., 2016), emotional well‐being (Tharani et al., 2017), and pre‐exam anxiety and stress experienced by the students (Hung et al., 2014; Hurd, 2007; Taylor & Fraser, 2013).

Due to technological developments, traditional components of learning environments have started to evolve. In this respect, most students have smart devices that can considerably alter audio and audiovisual stimuli extant in their learning environment (Dixit et al., 2020; Ranjbar et al., 2021). Thus, audio, and audiovisual stimuli might be essential dimensions of a learning environment. Despite the empirical evidence demonstrating the impact of audio and audiovisual stimuli on learning (Byun & Loh, 2015), and creativity (Alawad, 2012), ), researchers showed relatively less interest in the subtle effect of audio and audiovisual stimuli extant in a learning environment on students' autonomic responses. Moreover, audio and audiovisual stimuli in a learning environment have become more common during online‐distant education due to the Covid‐19 pandemic lockdown period. During this period, students could design their preferred atmosphere in terms of audio and audiovisual stimuli via smart electronic devices such as smartphones, tablets, consoles, computers, and TV. However, teachers lost their power to design an ideal learning environment in online‐distant education through Zoom or Microsoft Teams. Recent research investigating individuals' media usage behavior during the Covid‐19 lockdown supports our argument by documenting a dramatic increase in watching TV and internet usage (Dixit et al., 2020; Ranjbar et al., 2021). Online‐distant education, because of the Covid‐19 pandemic lockdown, did not include only online courses but also examinations. Many students took quizzes immediately after a particular class in their desired learning environment. Therefore, as audio and audiovisual stimuli influence students' autonomic nervous system (ANS) activity, which impacts several outcomes, such as learning and students' physical and mental well‐being, there is a need to understand the effect of learning environments containing different audio and audiovisual stimuli during an online academic examination.

The ANS controls many of the body's involuntary functions, including heart rate (HR), blood pressure, digestion, breathing, sexual arousal, papilla reflex, and stress responses (Gibbons, 2019; Hall & Hall, 2020). It has two main branches: the sympathetic nervous system (SNS) and the parasympathetic nervous system (PNS). The SNS is associated with the body's fight‐or‐flight response, while the PNS is responsible for relaxation. Accordingly, ANS might have a vital influence on students' learning experience and, more importantly, well‐being.

Due to the probable impact of background music on ANS, individuals could choose to listen to music while studying. Despite the popularity of pop music among people of different ages and nationalities while studying (Kotsopoulou & Hallam, 2010), research indicates that listening to classical music can have more positive effects on the ANS. Accordingly, studies have demonstrated that background Beethoven music can lead to improvements in heart rate, blood pressure, and mood (Darki et al., 2022). Additionally, Ellis and Thayer found that classical music may even stimulate dopamine release from the nucleus accumbens (Ellis & Thayer, 2010). Similarly, Menon and Levitin (2005) illustrated that passive listening to classical music resulted in significant activation of subcortical areas, including the nucleus accumbens, hypothalamus, ventral tegmental area, and insula, which are responsible for the regulation of autonomic and physiological responses to rewarding emotional stimuli. Researchers also have examined the effect of background classical music on academic performance. This line of research produced inconsistent results regarding the effect of background classical music on students' academic performance. For example, Dosseville et al. (2012) also found that classical music may aid learning in sports sciences students. However, Manthei and Kelly (1999) found that classical music did not affect math test scores. Moreover, from Kahneman's (1973) limited capacity viewpoint, music may place extra demands on cognitive resources' limiting capacity. Konečni (1982) also argued that listening to music may impair cognitive task performance as processing the music utilizes these finite cognitive resources. Therefore, whether background classical music can affect ANS activity while studying is unclear and needs to be clarified in well‐controlled experiments. At this point, we need to address the difference between the impact of music with lyrics and music without lyrics. Previous researchers have shown that the effect of background music on behavior and psychological outcome measures can vary depending on whether it has lyrics (Anderson & Fuller, 2010; de la Mora Velasco & Hirumi, 2020; Kämpfe et al., 2011; Shih et al., 2012). For instance, Shih et al. (2012) concluded that, if background music is played in the work environment, music without lyrics is preferable because songs with lyrics are likely to reduce worker attention and performance. For this reason, in the present study, we specifically examined the effect of nonlyrical classical music.

Background television may be the other most common way to create a learning environment used by students. Moreover, today almost every individual has access to smart devices that can act similar to television, such as digital video streaming platforms. More importantly, during the Covid‐19 lockdown period, the use of smart devices and televisions increased dramatically, especially in Western societies. Television broadcasts may transmit both visual and auditory information; hence, they may significantly impact students' autonomic responses. Moreover, the messages sent by a television broadcast include specific structural elements such as luminance, slow motion, cuts, animation, and music (Lang, 2000) that can place considerable extra demands on the information processing system. More importantly, television broadcasts also include a considerable amount of verbal content, which can be a severe irrelevant load for the verbal information processing.

In addition to the limited capacity approach mentioned earlier, the orienting response may be another valuable theoretical framework that can explain whether a learning environment's background sound characteristics may lead to variation in psychophysiological activity. The term orienting response describes the automatic allocation of information processing resources to the television program as a novel or exciting stimulus (Pool et al., 2003a). According to the orienting response approach, individuals orient their limited information processing resources toward a given novel stimulus, background television, in this instance (Lang, 2000). Further, a novel stimulus may constitute an extra load on cognitive resources and lead to specific psychophysiological responses to adapt the body for appropriate actions (Wang & Munoz, 2014). Hence, the effect of background television, as an extra load, in a learning environment on students' autonomic activity is of both theoretical and practical importance and deserves careful examination in a well‐controlled experiment.

It is widely recognized that cognitive workload affects the variability of autonomic activity. However, relying solely on one ANS marker may not provide sufficient insight into this connection. To gain a better understanding of the correlation between cognitive workload and ANS activity, it is best to measure several psychophysiological biomarkers that can indicate the performance of both the sympathetic and parasympathetic branches of ANS. In this regard, previous research provided evidence for the idea that heart rate (HR), heart rate variability (HRV), blood volume pulse amplitude (BVA), and skin conductance level (SCL) might reflect changes in ANS activity stemming from the cognitive workload, stressful events, anxiety, and worry (Critchley, 2002; Fitzgerald et al., 2022; Sousa et al., 2021; Thayer et al., 2012).

HRV is one of the noninvasive electrocardiographic (ECG) parameters that reflects the autonomic control of the heart (Heffernan et al., 2006; Miu et al., 2009) and arises from the interaction of the sympathetic and parasympathetic parts of the ANS (European Task Force, 1996). The SNS increases heart rate, constricts blood vessels, and decreases gastrointestinal motility (Aubert et al., 2003). Contrarily, the PNS decelerates heart rate and is associated with the digestive system (Aubert et al., 2003; Thayer & Lane, 2009). The heart rate of healthy persons displays beat‐to‐beat variations that result from fluctuations in ANS activity at the sinus node. HRV is represented by various measures, including time‐ and frequency‐domain metrics. To measure HRV, each normal‐to‐normal (NN) beat interval (R‐R in ECG) is determined in a continuous ECG record. Afterward, HRV time and frequency parameters are calculated using these successive NN beat intervals. The most widely used time‐domain parameters are the standard deviation of NN intervals (SDNN), the root mean square of successive NN interval differences (RMSSD), and the mean NN intervals (NNMean). The frequency‐domain parameters of the HRV, which are performed via fast Fourier transform (FFT), are another HRV measurement method and reflect the activity of the sympathetic and parasympathetic activities of the ANS. According to Massimo Pagani's model (Malliani et al., 1991; Pagani et al., 1986), the high‐frequency (HF) power (0.15−0.40 Hz) is seen primarily as a marker of cardiac parasympathetic tone (representing respiratory control mechanism). On the other hand, low‐frequency (LF) power (0.04−0.15 Hz) is considered a marker of cardiac sympathetic and parasympathetic activities (representing blood pressure and the thermoregulatory control mechanism). The low‐ to high‐frequency (LF/HF) ratio determines the balance between sympathetic and parasympathetic activity (del Paso et al., 2013). Antecedent studies demonstrated that HRV could reflect the heart's ability to adapt to stress induced by physiological, psychological, and environmental demands (Kim et al., 2018; Thayer et al., 2012). However, less is known about whether HRV may decrease due to learning environments with different auditory and audiovisual stimuli, especially when they are task irrelevant.

Another noninvasive indicator of the autonomic activity implemented in the present study is the blood volume pulse (BVP). BVP is considered a parameter that can reflect a peripheral pulse waveform converted to blood volume pulse amplitude (BVA) (Lin et al., 2015). According to Peper et al. (2007) and Shelley (2007), BVP is a parameter of arterial contraction and dilation controlled by the ANS. The sympathetic branch of the ANS contracts the microvessels of the finger, which leads to a decreasing level of BVA, while increased parasympathetic activity dilates the microvessels of the finger, which increases BVA (Lin et al., 2015; Shelley, 2007). Previous studies provided evidence that BVP might be associated with cognitive load (Zhou et al., 2017). In this respect, Zhou et al. (2017) demonstrated that BVP can make a distinction between the effect of high and low cognitive loads during a decision‐making task. BVP has also been found to be associated with emotional responses to musical stimuli (Salimpoor et al., 2009). However, it is still unclear whether BVA can reflect students' psychophysiological states in a learning environment with audio and audiovisual stimuli.

SCL is another widely used marker of the sympathetic branch of the ANS. SCL reflects only sympathetic activity. SCL refers to the variation of the electrical properties of the skin in response to sweat secretion (Benedek & Kaernbach, 2010). These glands are mainly located in the hypodermis of the palmar and plantar regions, and they generate sweat excreted through ducts (Groscruth, 2002. SCL reflects the output of integrated attentional affective and motivational processes within the central nervous system acting on the body (Critchley & Nagai, 2013). Psychophysiological aspects of the SCL stem from the reticular formation centers within the brainstem and thalamus (Luria & Homskaya, 1970). These subcortical mechanisms are also influenced by specific cortical regions; namely, the ventromedial prefrontal cortex, orbitofrontal cortex, left primary motor cortex, and the anterior and posterior cingulate, which have been shown to be associated with emotional and motivational behaviors (Critchley, 2002; Nagai et al., 2004).

In the present study, we aimed to explore the effect of background TV, classical music, and silence on students' autonomic responses, represented by HR, HRV, BVA, and SCL before, during, and after an experimental academic examination. To the best of our knowledge, no previous study tested whether learning environments with differing audio and audiovisual stimuli may lead to a variation in autonomic activity. Also, the present study implemented several standard psychophysiological biomarkers recorded from different anatomical regions (peripheral measures of the ANS). Therefore, the present study's results can provide an opportunity to better understand students' autonomic responses during a demanding academic task in the presence of differing audio and audiovisual stimuli.

In light of the theoretical explanations and research findings cited above, we predicted that the learning environment with background TV should increase HR. We also predicted that background TV reduces BVA and increases SCL levels. We further hypothesized that silence and background classical music do not affect individuals' HR, HRV, BVA, and SCL. In other words, classical music should act like silence during the mental task. We specifically reached this hypothesis due to above‐cited references showing the inconsistencies concerning the classical music's effect on ANS and mental performance. We also postulated that changes in HR, HRV, BVA, and SCL during the anticipation, challenge, and recovery should be greater in the background TV group.

## 2 METHODS

### 2.1 Participants

Seventy‐six first‐year undergraduate students (35 female) ranging in age from 19 to 24 (*M* = 21.7, *SD* = 2.8) participated in the study. All the participants took online courses and exams before the time of the study. Participants were required to abstain from using any medications or ergogenic aids that can affect the nervous and cardiovascular system functions and have no previously existing acute or chronic cardiovascular and psychiatric diseases. The local ethics committee approved all experimental procedures, participants signed informed consent before the study, and all data were collected following the latest version of the Helsinki Declaration.

### 2.2 Measures

#### 2.2.1 Measurement and psychophysiological data

A NeXus‐10 device, its supplied software of BioTrace^+^ (Mind Media CV; Roermond, Herten, the Netherlands), was used to measure all psychophysiological data. We recorded the HR, HRV, and BVA data via a BVP sensor (NX‐BVP1A) attached to the middle finger of the non‐dominant hand. The BVP monitors the relative blood flow in the hands (fingers) with near‐infrared light, a method known as photoplethysmography (PPG). Although ECG is the golden standard for measuring HRV, we preferred using a PPG sensor for a particular reason based on the issues reported by previous researchers (Lin et al., 2015; Peper et al., 2007; Pinheiro et al., 2016). That is to say, participants would experience an uncomfortable state because of ECG sensors, electrodes, gels, and electrodes' placement in the chest region, which can give rise to extra stress. Also, the PPG sensor allows the recording of multiple biosignals (HR, HRV, and BVA), reducing the number of sensors placed in participants’ bodies. We also measured the participant's tonic SCL. SCL was measured using a skin conductance sensor (NX‐GSR1A) at the index and ring finger of the non‐dominant hand. All psychophysiological data were recorded with a 24‐bit resolution at a frequency of 1024 Hz, except SCL (50 Hz notch filter).

#### 2.2.2 Analysis of HR, HRV, and data reduction

The HR and HRV of each participant were obtained based on the time series of peak‐to‐peak intervals immediately extracted from the BVP data. The HR and HRV are computed by taking the peak‐to‐peak distances between beats. Before analyses, we visually checked the normal‐to‐normal intervals (NN intervals), movement artifacts, and unusual heartbeats for artifact rejection. Afterward, we used the NeXus‐10 device‐supplied software BioTrace^+^ to correct the artifacts. In this regard, we used the “set artifact area and automatic artifact rejection‐interpolation” function of BioTRace^+^ software to obtain artifact‐free BVP data. Whenever an artifact area was determined, data of all channels at that particular time point was marked as artifacts and excluded from the analysis automatically. Corrected intervals (removed and interpolated) were converted into the interbeat intervals (IBI) time series. Artifact‐free BVP data were excluded from the statistical analysis when BVP artifacts were more than 10%. Finally, following the recommendations of the European Task Force (1996), the HRV for both the time‐ and frequency‐domain analyses was performed using the corrected IBI time series.

The HRV time domain was represented in the present study by the SDNN (ms) and the RMSSD (ms). We calculated the frequency‐domain parameters using the FFT algorithm (FFT window length = 512, Hanning window, 1024 points). As mentioned above, the primary HRV frequency‐domain indices are the LF (ms^2^) and HF power (ms^2^). The relationship between these two frequency bands (LF/HF ratio) was also analyzed.

#### 2.2.3 Blood volume amplitude analysis

The BVA was derived from the raw BVP signal via a PPG sensor and accepted to indicate changes in blood volume in arteries and capillaries. The PPG signal represents an average of all blood volume in the arteries and capillaries. The data were sampled at 1024 Hz and were expressed in millivolt (mV).

#### 2.2.4 Skin conductance level analysis

For the analysis, the mean SCL was determined over the experimental periods. Mean SCL was computed on BioTrace^+^ software for each of the 4 min periods. Measurements were made using the SC sensor with two disposable Ag/AgCl electrodes of 10 mm diameter that were placed on the distal pads of the index and ring fingers of the non‐dominant hand. The data were sampled at 32 Hz and stored on computer disks for subsequent analysis. Signals were expressed in micro Siemens (μS). The tonic component of the SCL was calculated as described in Boucsein (2012).

### 2.3 Experimental procedure

**FIGURE 1:**
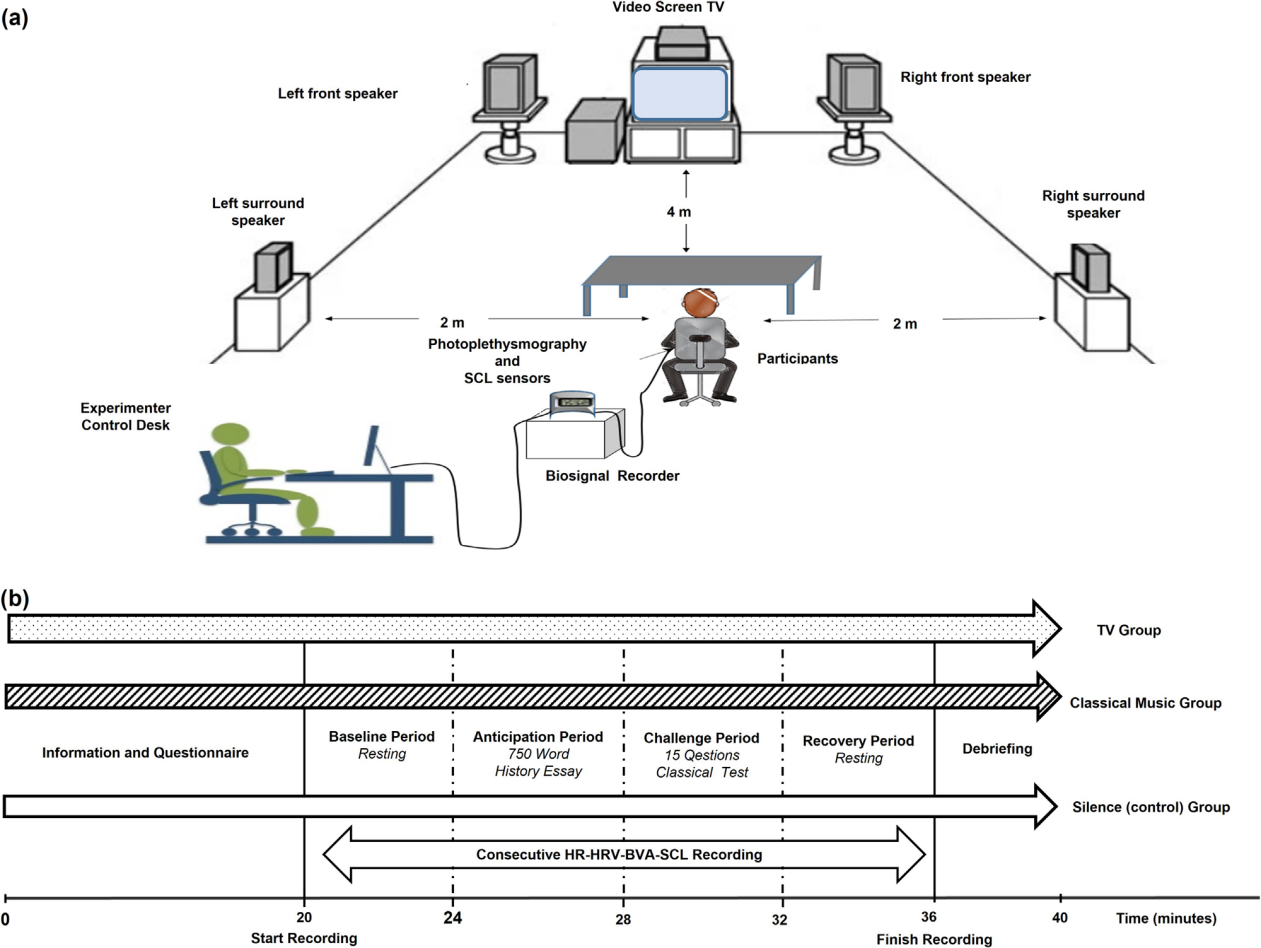
(a) Schematic representation of the experimental setup and (b) diagram of the experimental procedure.

The first author conducted all experiments in a particular room designed specifically for the present study (Figure 1a).

Participants were required to abstain from using medications affecting nervous system functioning for the study duration. Participants were told not to consume alcoholic drinks for 48 h and caffeinated drinks for 2 h before the experiment. Participants were also asked not to smoke for 2 h before the experiment. On arrival at the laboratory, participants were randomly assigned to one of the three groups: classical music (*n* = 24), background TV (*n* = 26), or silence group (*n* = 26). Then, participants gave informed consent. The individual participants were seated on a chair, and a PPG sensor was attached to the participant's non‐dominant hand middle fingers for HRV recording and SCL electrodes were attached to the same hand. The experimenter instructed the participants to position their non‐dominant hands as stable as possible. We also used an apparatus (silicon pad) to fix participants' non‐dominant hands to prevent movement artifacts. The experiment consisted of four consecutive periods, which lasted 4 min each for all the study groups, namely baseline, anticipation, challenge, and recovery (Figure 1b).

#### 2.3.1 Baseline period

In the experiment's first period, the participants' baseline autonomic indices were recorded for 4 min while sitting at rest. During this period, participants were asked not to talk and move.

#### 2.3.2 Anticipation period

At the beginning of the anticipation period, the experimenter explained the experimental procedure and the tasks. Accordingly, participants were told that there would be a history exam, and the individual exam scores would be publicized via faculty notice boards, intranet, and experimenters' social media accounts. Participants were then asked to choose one of the four identical envelopes containing 750‐word history essays. The participants were instructed to read and understand the 750‐word essay in the 4 min allocated while their autonomic responses were recorded. In this period, the classical music group was exposed to Johann Sebastian Bach's Orchestral Suite No. 3 in D major. The background TV group was exposed to the ATV channel's main news bulletin. We exposed participants to classical music and TV via an audio system placed at the four corners of the room, and a 127 cm TV placed 4 m in front of the participants, respectively. In both conditions, the sound level was 70 dBA. The silence group received no background sound intervention over the course of the entire experiment. Participants in all groups were not allowed to take notes during the anticipation period.

#### 2.3.3 Challenge period

At the end of the anticipation task, the experimenter took the essay back and gave the participant a standard examination paper consisting of 15 questions. Participants were instructed to answer all questions in the 4 min allocated while their autonomic responses were measured. Based on a pilot study of 10 students, we determined that it takes approximately 10 min to answer the 15 questions. As in the anticipation period, the classical music, background TV, and silence groups were exposed to the same background sound interventions during the challenge period.

#### 2.3.4 Recovery period

In the final period of the experiment, the experimenter ended the background sound intervention for the classical music and background TV groups, instructed participants to rest for 4 min, and subsequently recorded the autonomic responses during the recovery period.

### 2.4 Statistical analysis

To analyze the obtained data set, we first log‐transformed (Log 10) the HRV frequency‐domain parameters to satisfy linear analysis requirements. Then, we conducted a series of repeated measures ANOVAs to explore whether HR, HRV, BVA, and SCL responses differed significantly between the baseline, anticipation, challenge, and recovery conditions in the experimental groups with different sound environments. In reporting repeated measures ANOVA, we used a corrected degree of freedom via Greenhouse–Geisser estimates of sphericity if the assumption of sphericity was violated. Paired sample *t*‐tests with a Bonferroni correction were performed after a significant result of repeated measures ANOVA. In this respect, the standard confidence interval (.05) was divided by the number of *t*‐tests employed as post hoc. Therefore, the confidence interval was .0083 for the pairwise comparisons following a significant repeated measures ANOVA result. Lastly, we decided to examine whether psychophysiological indices recorded in the present study may differ significantly between the experimental groups via a series of one‐way ANOVAs. However, as the psychophysiological parameters, especially HRV frequency‐domain indices, might show large interindividual differences, we calculated the change scores of each psychophysiological parameter. Hence, we subtracted psychophysiological values recorded during anticipation, challenge, and recovery periods from their baseline values. Then, we used changes in these values during the anticipation, challenge, and recovery periods as dependent variables in the one‐way ANOVAs.

## 3 RESULTS

### 3.1 Results of the within‐subject analysis

#### 3.1.1 HR, HRV indices, BVA, and SCL changes in the background TV group

**FIGURE 2:**
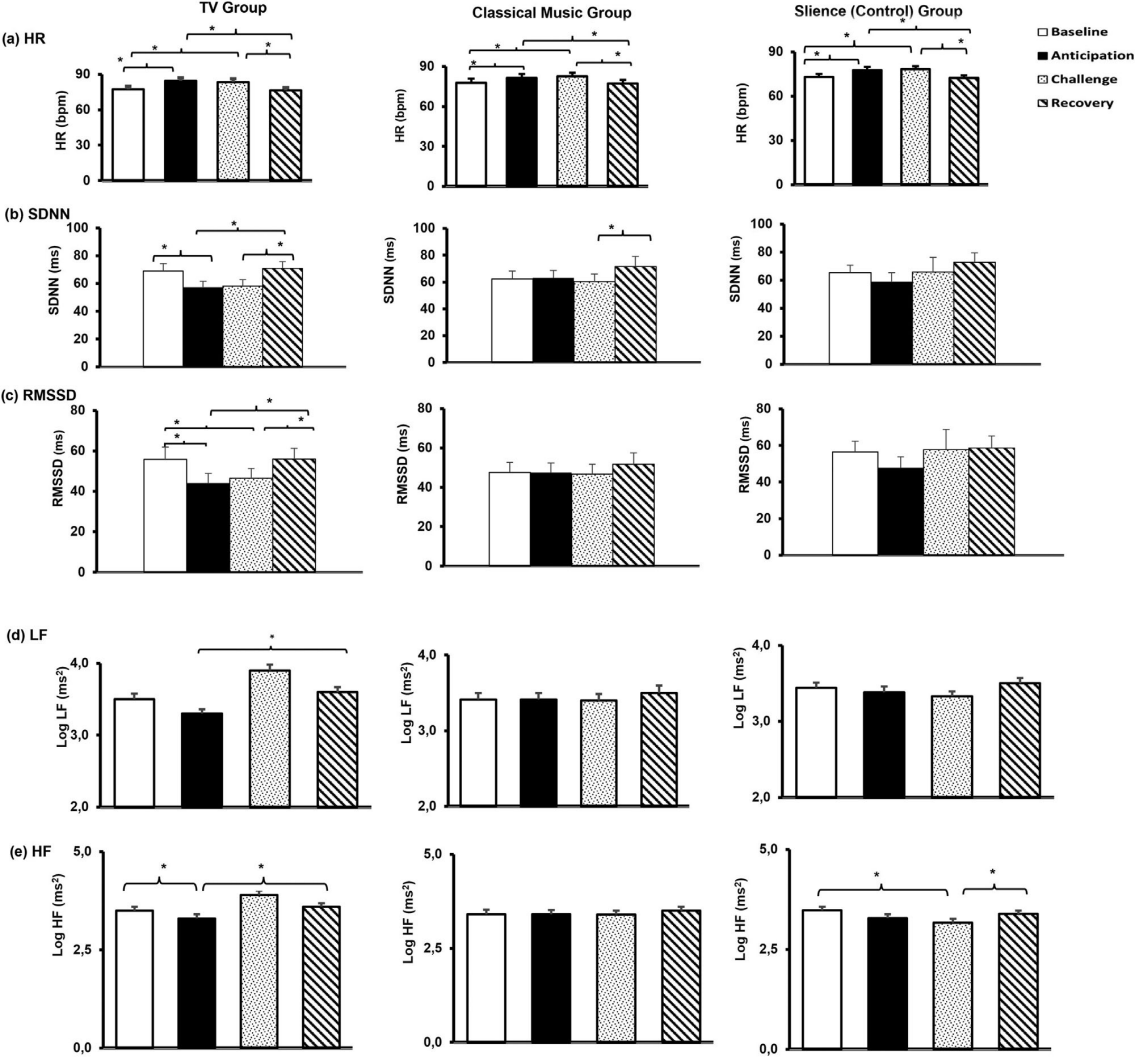
HR, SDNN, RMSSD, Log LF, and Log HF changes in all experimental groups. (a) HR changes, (b) SDNN, (c) RMSSD, (d) Log LF changes, (e) Log HF changes during the experiment for the background TV, classical music, and silence (control) group. *p < .008. Note that HRV time‐domain parameters decreased significantly during the anticipation and challenge periods in the background TV group, which indicates that sympathetic nervous system activity increased in the presence of audiovisual stimuli.

**FIGURE 3:**
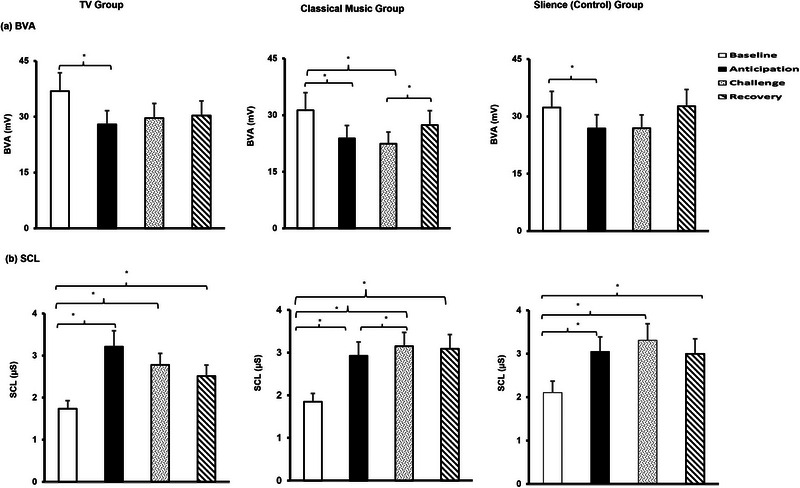
BVA and SCL changes in all experimental groups. (a) BVA changes, (b) SCL changes during the experiment for the background TV, classical music, and silence (control) group. *p < .008. Note that BVA decreased and SCL increased throughout the experiment, which indicates a parasympathetic withdrawal together with sympathetic activation.

The results of repeated measures ANOVA demonstrated that HR (F (3, 75) = 44.97, *p* = .001, *η* ^2^ = .65), SDNN (F (1.87, 46.83) = 9.15, *p* = .001, *η* ^2^ = .27), RMSSD (F (2.19, 54.94) = 13.33, *p* = .001, *η* ^2^ = .35), Log LF power (F (1.65, 41.25) = 5.09, *p* = .015, *η* ^2^ = .17), Log HF power (F (1.88, 41.18) = 7.88, *p* = .001, *η* ^2^ = .24) BVA (F (3, 75) = 6.29, *p* = .001, *η* ^2^ = .20) and SCL (F (1.44, 36.05) = 8.90, *p* = .002, *η* ^2^ = .26), differed significantly among the experimental conditions in the background TV group. However, the Log LF/HF ratio did not differ significantly among the experimental conditions in the background TV group (F (2.23, 55.88) = .884, *p* = .429, *η* ^2^ = .03). Table 1 summarizes both results of the repeated measures ANOVA and follow‐up paired sample *t*‐tests with Bonferroni correction as post hoc. Figure 2a indicates significant HR increases from baseline to anticipation and challenge periods. Figure 2a also depicts that HR during the recovery period was significantly lower than during anticipation and challenge periods, which means that HR was able to regress to its baseline level at the end of the experiment. HRV time‐domain parameters of SDNN and RMSSD significantly decreased from the baseline to the anticipation period (Figure 2b,c). Contrary to SDNN, the level of RMSSD continued to decrease from the baseline to the challenge period. SDNN and RMSSD were significantly lower during the recovery period than during the anticipation and challenge periods, which means, once again, HRV time‐domain parameters could regress to their baseline levels at the end of the experiment. Figure 2d shows the differences in Log LF power among experimental conditions in the background TV group. Accordingly, the Log LF power difference between anticipation and recovery was significant. Further, Log HF power decreased significantly from the baseline to the anticipation period (Figure 2e). In addition, Log HF power was significantly greater in the recovery than in the anticipation period. BVA decreased from baseline to anticipation and challenge conditions (Figure 3a). On the other hand, SCL increased from baseline to all experimental conditions (Figure 3b).

**TABLE 1:** The repeated measures ANOVA and multiple comparison tests with Bonferroni correction for autonomic indices in the background TV group.

	Repeated measures ANOVA										
	Baseline		Anticipation		Challenge		Recovery				
Variables	*Mean*	*SD*	*Mean*	*SD*	*Mean*	*SD*	*Mean*	*SD*	*F*	*p*	*ƞ* ^2^
HR (bpm)	77.33	12.75	84.52	13.47	83.49	14.22	76.49	11.55	46.4	.001*	.65
SDNN (ms)	68.82	23.99	57.01	23.7	58.2	23.21	70.85	25.24	9.15	.001*	.27
RMSSD (ms)	55.86	30.60	43.85	25.88	46.43	24.93	55.94	27.56	13.33	.001*	.35
Log LF (ms^2^)	3.5	0.04	3.3	0.30	3.9	0.33	3.6	0.34	5.09	.015*	.17
Log HF (ms^2^)	3.4	0.49	3.18	0.53	3.23	0.58	3.41	0.45	7.88	.001*	.24
Log LF/HF	0.11	0.34	0.18	0.30	0.16	0.31	0.19	0.38	0.884	.429	.03
BVA (mV)	36.86	25.35	27.93	18.91	26.93	19.95	30.33	19.96	6.29	.001*	.20
SCL (μS)	1.74	0.95	3.21	1.9	2.78	1.38	2.51	1.3	8.90	.002*	.26

	Multiple comparison tests with Bonferroni correction											
		a		b		c		d		e		f
	*t*	*p*	*t*	*p*	*t*	*p*	*t*	*p*	*t*	*p*	*t*	*p*
HR(bpm)	−7.93	.001*	−6.46	.001*	1.15	.261	1.24	.224	9.88	.001*	8.35	.001*
SDNN (ms)	3.59	.001*	2.34	.027	−.828	.416	0.460	.649	−4.71	.001*	−3.38	.001*
RMSSD (ms)	3.90	.003*	3.32	.003*	−.039	.969	−1.36	.186	−5.66	.001*	−4.01	.001*
Log LF (ms^2^)	2.05	.050	1.43	.164	−2.62	.015	−0.439	.664	−3.46	.002*	−2.41	.023
Log HF (ms^2^)	4.11	.001*	2,.17	.039	−0.207	.838	−0.845	.406	−4.65	.001*	−2.56	.017
BVA (mV)	3.55	.002*	2.73	.011	2.79	.010	−1.08	.287	−1.22	.232	−344	.734
SCL (μS)	−5.00	.001*	−5.74	.001*	−3.44	.002*	1.09	.284	1.69	.104	1.94	.063

* *p* < .0083.

Abbreviations: a, comparison of baseline and anticipation conditions; b, comparison of baseline and challenge conditions; c, comparison of baseline and recovery conditions; d, comparison of anticipation and challenge conditions; e, comparison of anticipation and recovery conditions; f, comparison of challenge and recovery conditions.

#### 3.1.2 HR, HRV indices, BVA, and SCL changes in the classical music group

The repeated measures ANOVA revealed that HR (F (1.91, 44.01) = 11.36, *p* = .001, *η* ^2^ = .33), SDNN (F (3, 69) = 3.90, *p* = .012, *η* ^2^ = .15), BVA (F (1.82, 41.85) = 8.36, *p* = .001, *η* ^2^ = .26), SCL (F (1.41, 32.59) = 21.69, *p* = .001, *η* ^2^ = .49) differed significantly among the experimental conditions in the classical music group. On the other hand, Log LF power (F (3, 69) = 786, *p* = .506, *η* ^2^ = .03), Log HF power (F (2.21, 50.84) = .119, *p* = .905, *η* ^2^ = .005), Log LF/HF ratio (F (2.25, 51.77) = .783, *p* = .476, *η* ^2^ = .02), and RMSSD (F (2.02, 46.45) = 1.23, *p* = .303, *η* ^2^ = .05] did not differ significantly among experimental conditions. Table 2 summarizes both results of the repeated measures ANOVA and follow‐up paired sample *t*‐tests with Bonferroni correction as post hoc. Figure 2a indicates that HR significantly increased from baseline to anticipation and challenge conditions. However, HR decreased significantly from the anticipation and challenge periods to recovery periods. HRV parameters remained almost stable during the entire experiment except for SDNN (Figure 2b,c). Hence, SDNN in the classical music group increased significantly from the challenge to the recovery period. Figure 3a shows that BVA decreased from baseline to anticipation and challenge conditions significantly. Contrarily, BVA significantly increased from challenge to recovery (Figure 3a). SCL significantly increased from baseline to anticipation, challenge, and recovery periods (Figure 3b).

**TABLE 2:** The repeated measures ANOVA and multiple comparison tests with Bonferroni correction for autonomic indices in the classical music group.

	Repeated measures ANOVA										
		Baseline		Anticipation		Challenge		Recovery			
Variables	*Mean*	*SD*	*Mean*	*SD*	*Mean*	*SD*	*Mean*	*SD*	*F*	*p*	*ƞ* ^2^
HR (bpm)	76.86	15.44	81.56	14.33	82.57	14.14	77.39	12.18	11.36	.001*	.33
SDNN (ms)	62.48	28.46	63.12	26.67	60.48	27.27	71.62	36.58	3.90	.012*	.15
RMSSD (ms)	45.56	25.48	47.42	24.39	46.75	24.19	51.28	28.32	1.23	.303	.05
Log LF (ms^2^)	3.41	0.42	3.41	0.43	3.40	0.41	3.5	0.46	0.786	.506	.03
Log HF (ms^2^)	3.25	0.56	3.27	0.52	3.2	0.51	3.2	0.53	0.119	.905	.005
Log LF/HF	0.15	0.40	0.14	0.40	0.17	0.35	0.23	0.43	0.783	.476	.33
BVA (mV)	31.29	22.90	23.82	16.70	22.45	15.05	27.37	18.51	8.39	.001*	.26
SCL (μS)	1.84	0.96	2.02	1.59	3.14	1.59	3.08	1.63	21.69	.001*	.49

		Multiple comparison tests with Bonferroni correction										
		a		b		c		d		e		f
	*t*	*p*	*t*	*p*	*t*	*p*	*t*	*p*	*t*	*p*	*t*	*p*
HR (bpm)	−4.86	.001*	−4.61	.001*	−0.322	.750	−1.26	.219	3.09	.005*	5.18	.001*
SDNN (ms)	−0.246	.808	0.653	.553	−2.33	.028	0.851	.404	−2.11	.046	−3.09	.005*
BVA (mV)	3.02	.006*	3.6	.002*	1.8	.086	1.4	.175	−2.13	.043	−3.63	.001*
SCL (μS)	−4.29	.001*	−5.43	.001*	−5.03	.001*	−3.87	.001*	−1.33	.195	0.623	.536

* *p* < .0083.

Abbreviations: a, comparison of baseline and anticipation conditions; b, comparison of baseline and challenge conditions; c, comparison of baseline and recovery conditions; d, comparison of anticipation and challenge conditions; e, comparison of anticipation and recovery conditions; f, comparison of challenge and recovery conditions.

#### 3.1.3 HR, HRV indices, BVA, and SCL changes in the silence group

The repeated measures ANOVA indicated that HR (F(3, 75) = 25.07, *p* = .001, *η* ^2^ = .50), Log HF (F (2.26, 56.61) = 9.33, *p* = .001, *η* ^2^ = .27), Log LF/HF ratio (F (3, 75) = 3.20, *p* = .025, *η* ^2^ = .11), BVA (F(2.21, 64.15) = 5.84, *p* = .004, *η* ^2^ = .17), and SCL (F(1.76, 44.21) = 14.54, *p* = .001, *η* ^2^ = .35) were significantly different among the experimental conditions in the silence. However, SDNN (F (1.38, 34.56) = .809, *p* = .412, *η* ^2^ = .03), and RMSSD (F (1.25, 31.44) = .676, *p* = .450, *η* ^2^ = .03), Log LF (F (1.99, 49.94) = 2.38, *p* = .103, *η* ^2^ = .09) did not changed significantly among the experimental conditions. Table 3 summarizes both results of the repeated measures ANOVA and follow‐up paired sample *t*‐tests with Bonferroni correction as post hoc for the silence group. Figure 2a indicates that HR significantly increased from baseline to anticipation and challenge conditions. However, HR decreased significantly from the anticipation and challenge periods to recovery periods. HRV time‐domain parameters remained unchanged during the entire experiment (Figure 2b,c). On the contrary, Log HF decreases from baseline to the challenge were statistically significant. Further, Log HF increased from the challenge to the recovery period in the silence group (Figure 2e). Note that despite the significant main effect of the within‐subject factor Log LF/HF ratio, paired sample *t*‐tests with Bonferroni corrections did not reveal significant Log LF/HF ratio differences among the experimental conditions in the silence group. Figure 3a shows that BVA decreased significantly from baseline to anticipation condition and did not change among other experimental conditions. SCL increased from baseline to anticipation, challenge, and recovery conditions (Figure 3b).

**TABLE 3:** The repeated measures ANOVA and multiple comparison tests with Bonferroni correction for autonomic indices in the silence (control) group.

		Repeated measures ANOVA									
		Baseline		Anticipation		Challenge		Recovery			
Variables	*Mean*	*SD*	*Mean*	*SD*	*Mean*	*SD*	*Mean*	*SD*	*F*	*p*	*ƞ* ^2^
**HR (bpm)**	72.94	10.4	77.67	10.48	78.22	10.63	72.26	9.69	25.07	**.001** *	.50
**SDNN (ms)**	65.21	27.23	59.0	32.33	65.78	53.66	72.78	33.85	0.809	.412	.03
**RMSSD (ms)**	56.49	30.15	47.74	30.44	57.66	56.45	58.69	33.22	0.676	.450	.03
**Log LF (ms^2^)**	3.44	0.39	3.38	0.44	3.33	0.36	3.5	0.39	2.38	.103	.09
**Log HF (ms^2^)**	3.48	0.49	3.28	0.56	3.17	0.51	3.39	0.46	9.33	**.001** *	.27
**Log LF/HF**	−0.002	0.35	0.10	0.31	0.16	0.33	0.12	0.32	3.29	**.025** *	.11
**BVA (mV)**	33.31	21.51	26.81	18.48	26.89	17.77	32.66	22.47	4.02	**.022** *	.14
**SCL (μS)**	2.10	1.36	3.05	1.71	3.31	1.93	2.99	1.78	14.54	**.001** *	.37

	Multiple comparison tests with Bonferroni correction											
	a		b		c		d		e		f	
	*t*	*p*	*t*	*p*	*t*	*p*	*t*	*p*	*t*	*p*	*t*	*p*
**HR (bpm)**	−5.70	**.001** *	−6.11	**.001** *	0.756	.457	−0.671	.509	5.01	**.001** *	8.17	**.001** *
**Log HF (ms^2^)**	2.32	.029	4.30	**.001** *	1.24	.224	2.19	.039	−1.85	.076	−4.95	**.001** *
**Log LF/HF**	−1.70	.101	−2.85	.009	−2.52	.018	−1.27	.216	−0.363	.720	0.732	.471
**BVA (mV)**	2.91	**.007** *	2.67	.013	−0.123	.903	−0.063	.950	−2.03	.053	−2.30	.030
**SCL (μS)**	−8.88	**.001** *	−5.25	**.001** *	−3.73	**.001** *	−1.38	.178	0.242	.811	2.07	.048

* *p* < .0083.

Abbreviations: a, comparison of baseline and anticipation conditions; b, comparison of baseline and challenge conditions; c, comparison of baseline and recovery conditions; d, comparison of anticipation and challenge conditions; e, comparison of anticipation and recovery conditions; f, comparison of challenge and recovery conditions.

### 3.2 Results of the between‐subject analysis

**FIGURE 4:**
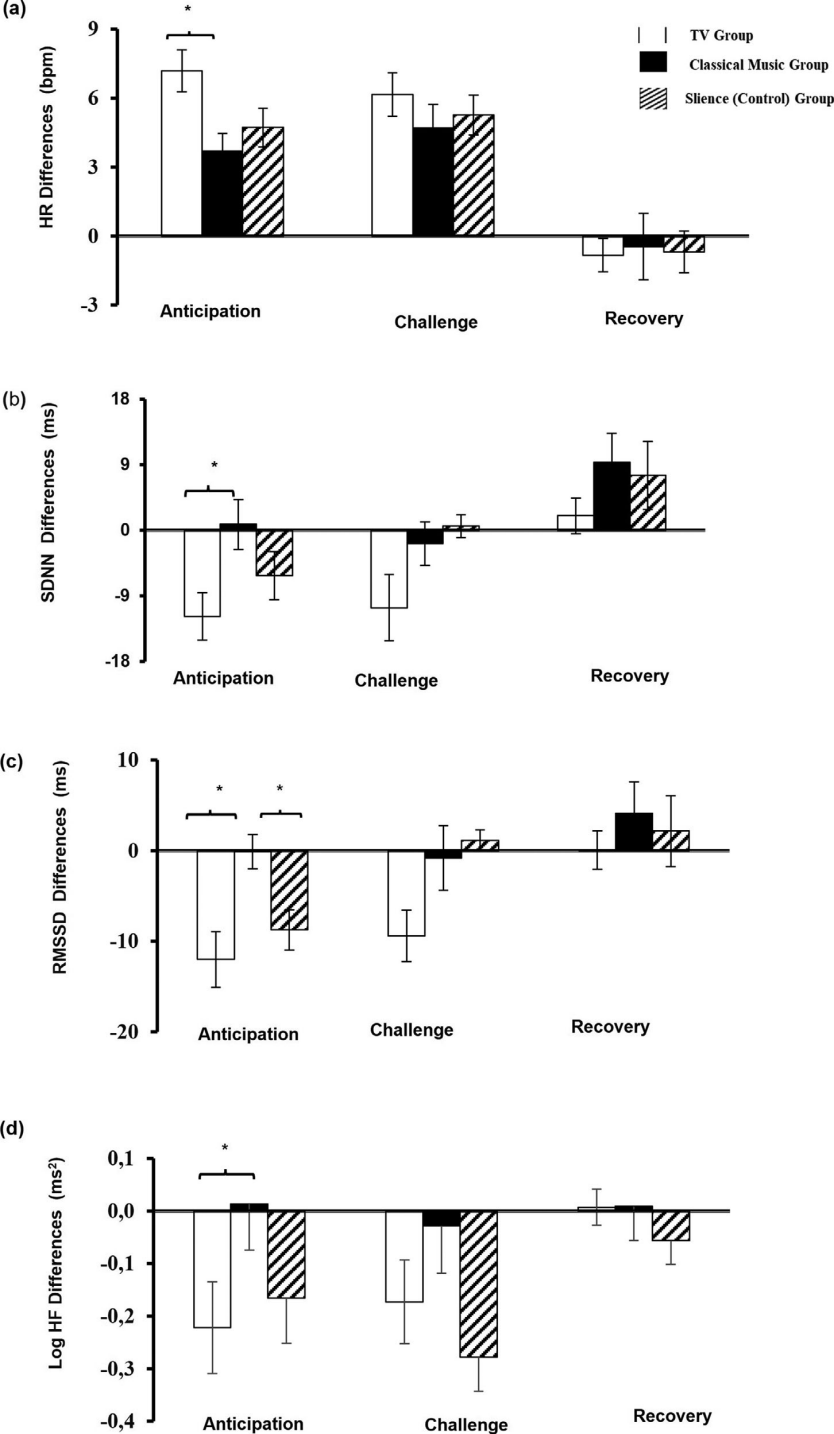
HR, SDNN, RMSSD, and Log HF changes in all experimental groups. (a) HR changes, (b) SDNN, (c) RMSSD, (d) Log HF changes during the experiment for the background TV, classical music, and silence (control) group. *p < .05. Note that changes in psychophysiological parameters during the anticipation and challenge periods were more remarkable in the background TV group.

In addition to within‐subject comparison, we tested whether changes in physiological parameters during anticipation, challenge, and recovery differed significantly among the experimental groups. For this purpose, we first calculated how much physiological parameters deviated from their baseline measures. Then, we performed a series of one‐way ANOVAs to explore whether changes in these values differed between the background TV, classical music, and silence groups. As illustrated in Table 4, the results of the one‐way ANOVAs revealed that HR (F(2, 73) = 4.57, *p* = .013), SDNN (F(2, 73) = 3.56, *p* = .033), RMSSD (F(2, 73) = 6.00, *p* = .004), and Log HF power (F(2, 73) = 3.95, *p* = .024) changes during the anticipation period differed significantly among the experimental groups. Log LF power, Log LF/HF ratio, BVA, and SCL changes during the anticipation period were not statistically significant. As shown in Table 4, no other significant changes in psychophysiological parameters were observed during the anticipation period. Results of the one‐way ANOVA also showed that changes in all psychophysiological indices during the challenge and recovery periods were not statistically significant. Figure 4 demonstrates the results of the post hoc Tukey tests following significant results of the one‐way ANOVA. Accordingly, the results of the Tukey test showed that HR changes were more remarkable in the background TV group (7.19 bpm) than in the classical music group (3.7 bpm) during the anticipation period (Figure 4a). Similarly, SDNN changes during the anticipation period differed significantly between the background TV and classical music groups. Examination of the descriptive statistics concerning changes in SDNN revealed that while there was an 11.8 ms decrease in the background TV group, SDNN increased by 0.84 ms in the classical music group during the anticipation period (Figure 4b). The post hoc Tukey test results showed a significant difference regarding the changes in RMSSD during the anticipation period. RMSSD changes in the background TV group were significantly greater than in the classical music group (Figure 4c). Hence, RMSSD decreased by 12 ms in the background TV group, while RMSSD decreased only by 0.13 ms in the classical music group. Moreover, the post hoc Tukey test also indicated that changes in RMSSD during the anticipation period significantly differed between the classical music (0.13 ms) and silence (8.7 ms) groups. Lastly, the post hoc Tukey test showed significant differences in Log HF power changes during the anticipation period. Thus, Log HF power decreased in the background TV group by 0.222 ms^2^ and increased in the classical music group by 0.013 ms^2^ (Figure 4d).

**TABLE 4:** One‐way ANOVA of change scores of psychophysiological indices among the anticipation, challenge, and recovery periods.

Variables	Change scores	Groups	*Mean*	*SD*	*F*	*p*
HR (bpm)	Anticipation	Background TV	7.19	4.62	4.57	.013*
		Classical music	3.70	3.73		
		Silence	4.72	4.22		
	Challenge	Background TV	6.16	4.85	0.596	.554
		Classical music	4.71	5		
		Silence	5.27	4.39		
	Recovery	Background TV	−0.83	3.71	0.032	.969
		Classical music	−0.46	7.06		
		Silence	−0.68	4.61		
SDNN (ms)	Anticipation	Background TV	−11.80	16.73	3.56	.033*
		Classical music	0.84	16.71		
		Silence	−6.20	16.82		
	Challenge	Background TV	−10.62	23.10	0.671	.514
		Classical music	−1.80	14.65		
		Silence	0.57	56.41		
	Recovery	Background TV	2.02	12.49	1.002	.372
		Classical music	9.33	19.56		
		Silence	7.56	23.78		
RMSSD (ms)	Anticipation	Background TV	−12.00	15.67	6.002	.004*
		Classical music	−0.13	9.21		
		Silence	−8.74	11.34		
	Challenge	Background TV	−9.42	14.44	0.641	.530
		Classical music	−0.81	17.52		
		Silence	1.16	56.91		
	Recovery	Background TV	0.08	10.79	0.381	.685
		Classical music	4.12	17.13		
		Silence	2.19	19.94		
Log LF (ms^2^)	Anticipation	Background TV	−0.149	0.36	0.902	.410
		Classical music	0.006	0.37		
		Silence	−0.062	0.47		
	Challenge	Background TV	−0.125	0.44	0.593	.556
		Classical music	−0.003	0.35		
		Silence	−0.111	0.47		
	Recovery	Background TV	0.092	0.18	0.033	.968
		Classical music	0.091	0.35		
		Silence	0.073	0.36		
Log HF (ms^2^)	Anticipation	Background TV	−0.222	0.27	3.94	.024*
		Classical music	0.013	0.26		
		Silence	−0.166	0.36		
	Challenge	Background TV	−0.173	0.40	2.51	.088
		Classical music	−0.028	0.44		
		Silence	−0.278	0.33		
	Recovery	Background TV	0.007	0.17	0.585	.560
		Classical music	0.009	0.32		
		Silence	−0.056	0.23		
LF/HF	Anticipation	Background TV	−0.070	0.29	0.847	.433
		Classical music	0.005	0.31		
		Silence	−0.107	0.32		
	Challenge	Background TV	−0.044	0.33	1.35	.265
		Classical music	−0.028	0.36		
		Silence	−0.169	0.30		
	Recovery	Background TV	−0.081	0.23	0.311	.734
		Classical music	−0.083	0.22		
		Silence	−0.128	0.25		
BVA (mV)	Anticipation	Background TV	8.927	12.79	0.575	.565
		Classical music	7.472	12.10		
		Silence	5.497	10.33		
	Challenge	Background TV	7.233	13.51	0.506	.605
		Classical music	8.841	12.09		
		Silence	5.417	10.33		
	Recovery	Background TV	6.531	11.90	2.037	.138
		Classical music	3.920	10.56		
		Silence	−0.345	14.28		
SCL (μS)	Anticipation	Background TV	−1.47	1.50	1.43	.244
		Classical music	−1.07	1.22		
		Silence	−0.94	0.54		
	Challenge	Background TV	−1.04	0.92	0.361	.698
		Classical music	−1.29	1.14		
		Silence	−1.21	1.17		
	Recovery	Background TV	−0.77	1.14	1.01	.370
		Classical music	−1.23	1.20		
		Silence	−0.90	1.22		

* *p* < .05.

## 4 DISCUSSION

The present study investigated whether a learning environment with a background TV, classical music, or just silence may affect students' ANS activity represented by HR, HRV, BVA, and SCL during and after an experimental academic examination. We found that HR, BVA, and SCL differed significantly among the experimental conditions in all groups. In contrast, HRV time‐domain parameters showed significant differences in the group with the background TV. This group had to process more cognitive information such as audio, audiovisual stimuli, and verbal content. However, HRV parameters remained relatively stable in the groups that listened to classical music or experienced silence. The data partially supported our hypothesis concerning differences in changes in psychophysiological parameters during academic examination. Thus, differences in HR and HRV were more remarkable in the background TV group only during the anticipation period.

In previous studies, researchers focused primarily on whether a learning environment with a background TV might influence homework performance or achievement (Pool et al., 2000, 2003b). To the best of our knowledge, no previous study investigated the effect of the learning environment with background TV on students' psychophysiological responses, in this case, HRV, BVA, and SCL. Therefore, the first finding we want to discuss is the influence of 8 min of background TV exposure on autonomic responses represented by HR, HRV time‐ and frequency‐domain indices, BVA and SCL. Hence, as the higher level of HRV is associated with functionally efficient autonomic adaptation to environmental demands (Laborde et al., 2017; Shaffer & Ginsberg, 2017), our results documented the decrement in HRV time‐ and frequency‐domain parameters indicating that background TV inhibited the autonomic adaptation of students. Specifically, a decrement in RMSSD and Log HF power, which is widely accepted as a marker of the PNS (Shaffer & Ginsberg, 2017) indicates a parasympathetic withdrawal in the presence of the background TV. Cognitive load theory (Sweller, 1994), focusing on the limited capacity of working memory, which is also closely related to attention (Oberauer, 2019), might explain autonomic changes in the background TV group. In this respect, we attribute the HRV decrement observed in the background TV group to the intensive cognitive load due to the visual and auditory stimuli as well as verbal content from the TV. In contrast to the classical music used in the study, the TV playing in the background contained much verbal content that could stimulate verbal processing networks. Accordingly, it appears that TV's auditory, visual, and verbal stimuli functioned as an extraneous cognitive load, as proposed by Sweller et al. (2019), that does not contribute to or even impair the limited capacity of the working memory. As the psychophysiological approach can reflect cognitive load (Chen et al., 2016), we concluded that impairment in HRV time‐domain indices might be due to the TV's unnecessary auditory, visual, and verbal stimuli. Another study by Larmuseau et al. (2020), using a very similar experimental design to our study, reported that HR and skin temperature might have the potential to assess cognitive load. In addition, former research illustrated the link between vagal cardiac tone and selective attention to neutral stimuli under load (Park et al., 2013). Load theory of attention (Lavie, 2005; Lavie et al., 2004) asserts that processing task‐relevant information might play a role in the processing of task‐irrelevant information, which could be another framework that possibly explains our results. In this regard, previous research from the perspective of the load theory of attention demonstrated that working memory load might increase task‐irrelevant information interference (Lavie et al., 2004). Moreover, an excessive cognitive load that exceeds the optimal level and associated stress might lead to attentional narrowing and distractibility (Bong et al., 2016), which in turn gives rise to the suppression of parasympathetic activity. Because the experimental task used in the present study requires verbal processing resources, it is logical to expect elevated sympathetic activity due to the background TV, which includes verbal messages alongside audiovisual stimuli.

The results also demonstrated a decrease in BVA, which reflects arterial dilation and contraction that are controlled by autonomous nervous systems (Lin et al., 2015; Shelley, 2007) in the background TV group. Hence, these results provided additional support for the argument that background TV existing in the learning environment may give rise to parasympathetic withdrawal. SCL changes observed in the background TV group also indicate an increase in sympathetic arousal. Accordingly, SCL increased significantly during the anticipation and challenge periods in the background TV group. Taken together, considering the changes in ANS markers in the background TV group, it seems that task‐irrelevant audiovisual stimuli or unnecessary cognitive loads existing in a learning environment might suppress the parasympathetic activation and promote sympathetic activity.

Another critical issue that we want to highlight is the progression of HRV responses to background TV intervention. As illustrated in Figure 2b,c, neither the SDNN nor RMSSD was able to regress to its baseline levels during the 8‐minute‐long anticipation and challenge periods in the background TV group. In other words, participants' ANS failed to habituate to background TV while engaging in a task that requires considerable mental effort. Besides, findings from previous studies and the present studies contribute to the argument suggesting that individuals might not develop an adaption to certain distracting stimuli (Carter & Beh, 1989; Evans et al., 1998; Trimmel et al., 2012). Contrarily, HR, BVA, and SCL could relatively regress to their baseline level.

In contrast to the HRV decrement observed as a result of the within‐subject comparisons in the background TV group, HRV time‐ and frequency‐domain indices remained almost stable in the background classical music and silence group. These results provided preliminary support for the view that the HRV decrement observed in the background TV group was due to individuals' efforts devoted to filtering out the irrelevant stimulus generated by the TV. It is important to note that the results showed a remarkable vagal withdrawal in the group with background TV. These results could be attributed to the presence of verbal stimuli, which were absent in the groups listening to classical music and experienced silence. Hence, the lyrics of musical pieces might influence how music affects psychophysiological activity during a demanding mental task.

As stated earlier in this paper, individuals may believe that listening to classical music while studying lessons may benefit a better learning outcome. However, functional MR and PET studies showed that brain regions associated with the control of attention, memory, working memory, emotion, and the limbic system also engage in processing musical auditory information (Salimpoor et al., 2013; Trost et al., 2012). Hence, one could suggest that background classical music may put an extra demand on the information processing capacity during a mental task, which, in turn, may decrease HRV. Interestingly, however, classical music did not influence HRV during the anticipation (reading text) and challenge (examination) conditions. From a different point of view, classical music inhibited a possible decrement in HRV during the anticipation and challenge conditions. Moreover, our between‐subject comparisons concerning the changes in HR and HRV documented that while background TV led to an increase in HR and a decrease in HRV, classical music inhibited the impairment in the same indices. Barbosa et al. (2014) also reached similar conclusions revealing that classical music does not affect HRV time‐ and frequency‐domain indices during a mental task. A recent study by Jäncke et al. (2018) may provide a theoretical base for the lack of HRV change observed in the classical music group. Accordingly, these authors found event‐related desynchronizations as a result of listening to Mozart's music during attentive (paying attention to the music) and passive listening (paying more attention to the film sequence) conditions. Event‐related desynchronization represents the activation of the superior parietal lobule, the intraparietal sulcus area, and parts of the dorsal visual cortex, which are responsible for selective attention (Sadaghiani et al., 2010). It appears that certain classical music pieces, Johann Sebastian Bach's Orchestral Suite No. 3 in D major in this case, have the potential to recruit the neural pathways associated with selective attention. Therefore, we concluded that participants could direct their attention to the tasks at hand in the presence of background classical music especially with no lyrics. In other words, background classical music during a mental task might have some beneficial effect or at least no discernible detrimental effect. Inconsistent results suggested there might be some other factor that can moderate the link between classical music and ANS activity. As such, a study by Latha et al. (2015) showed that classical music might give rise to a more evident increase in females' parasympathetic activity.

Contrary to HRV parameters, HR, and SCL increased and BVA decreased significantly during anticipation and challenge periods in the classical music and silence groups, which means that sympathetic activity has been promoted by our intervention. These results indicated that HRV might be influenced by the severe audiovisual stimulus in contrast to HR, BVA, and SCL. Taken together, based on the results of the present study, especially the decrement in RMSSD and Log HF, we concluded that background TV is a severe stressor that can alter autonomic activity. Moreover, the current study results supported the view that HRV is a valid indicator of psychological and environmental stress, emotional states, and mental challenge (Appelhans & Luecken, 2006; Shaffer & Ginsberg, 2017; Von Borell et al., 2007).

The last finding we want to discuss is the relatively stable progress of the LF/HF ratio in all experimental groups. Although our results, in general, indicated a parasympathetic withdrawal and sympathetic elevation, LF/HF ratio which is accepted as sympathovagal balance indice (Laborde et al., 2017) did not change due to the experimental manipulations. We offer several explanations for observed results documenting the relatively stable progress of the LF/HF ratio in our experiment. First, psychophysiological parameters, LF/HF ratio, in this case, might not reflect ANS activity in all circumstances. Second, certain individual differences might play an important role in understanding psychophysiological responses to stress. In this respect, a previous study by (Laborde et al., 2011) found an increase in LF/HF ratio as a result of emotional manipulation only in individuals with low trait emotional intelligence. More importantly, researchers aiming to examine the effect of stress on sympathovagal balance should consider the possibility that LF/HF ratio might not reflect the sympathovagal balance (Billman, 2013). According to Billman (2013), due to the poor relationship between LF power and sympathetic nerve activation LF/HF ratio may not reflect sympathovagal balance accurately.

This novel study may have some implications for both practitioners and researchers. First, future researchers aiming to understand the effect of background distracting audio and audiovisual stimulus on ANS activity should consider the possibility that HR, HRV, BVA, and SCL are not equally sensitive to distracting stimuli during a demanding mental task. Therefore, we recommend recording several standard psychophysiological biomarkers for a better understanding of the issue. Further, considering the possibility that different participants might tend to respond to stress by activating different major physiological systems (Lacey, 1967) researchers are recommended to implement multiple ANS markers in psychophysiological research. Moreover, the experimental task used in the present study required primarily linguistic skills, and future studies might use experimental tasks requiring mathematical skills. Students, teachers, and parents should consider the possibility that TV existing in a learning environment might lead to elevated psychological and physiological stress responses. Lastly, classical music existing in a learning environment might not be a distractor. Rather, classical music might prevent sympathetic activation and parasympathetic withdrawal during a demanding mental task. Further, it appeared that classical music might activate the neural pathways associated with selective attention. However, bearing in mind the possibility that the effect of background music may rely on whether it has lyrics (Anderson & Fuller, 2010; Shih et al., 2012).

The present study has some limitations. In the present study, we did not measure perceived stress in addition to objectively measured stress. Also, we did not measure participants’ perceived state anxiety and difficulty concerning the task. Our study focused solely on the influence of classical music without lyrics in the background. However, previous research indicates that the effect of background music may vary depending on whether it includes lyrics. Therefore, future studies should explore the effects of background music with and without lyrics. Finally, in this study, respiration activity was not monitored and controlled. So, given that the breathing pattern may affect HRV, especially HF power (due to respiratory sinus arrhythmia) (Quintana & Heathers, 2014), future studies might consider taking under control the effect of breathing on HRV.

## 5 CONCLUSION

Results of the present study indicated that classical music and silence gave rise to almost similar responses in terms of recorded psychophysiological biomarkers which means that classical music has at least null effects during a mental task. Rather, a certain type of music especially calm music with no lyrics might speed up tonic vagal activity and increase autonomic regulation capacity in reading comprehension tasks. Students might benefit from Johann Sebastian Bach's classical music composition of Orchestral Suite No. 3 in D major while engaging in a demanding reading comprehension task. We also concluded that the impact of music or other background audio and audiovisual stimuli may be influenced by the presence of verbal contents in those stimuli. In this respect, the background TV having audio, audiovisual stimuli, and more critically verbal content that can interfere with reading tasks might suppress vagal activity. Finally, the HR, HRV, BVA, and SCL methods are not equally sensitive mediums to indexing ANS activity in a learning environment. In this regard, HRV may represent students' sympathetic and parasympathetic activity only when they are exposed to severe stimuli during a cognitive task. Studies executed with SCL and BVA may give more precise results if performed with HRV.

## AUTHOR CONTRIBUTIONS

İlker Balıkçı, Serdar Tok, and Erdal Binboğa were all involved in the design, writing, and editing of the study and manuscript. All authors approved the final version for submission.

## CONFLICT OF INTEREST STATEMENT

The authors declare no conflicts of interest.

## FUNDING INFORMATION

The authors did not receive support from any organization for the submitted work.

### PEER REVIEW

The peer review history for this article is available at https://publons.com/publon/10.1002/brb3.3153

## Data Availability

The datasets generated during and/or analyzed during the current study are available from the corresponding author on reasonable request.
